# A New Splint for Fractures of the Jaw

**Published:** 1862-10

**Authors:** J. F. Locke


A New Splint for Fractures of the Jaw.—*By J. F. Locke, M. D.*—So numerous are the cases in the army of fractures of the lower jaw from the musket ball, that I am induced to present, through your journal, to its readers, a method which I have used for the treatment of such cases, and first made use of in April, 1857.

I found, on examination of that patient, that there was a fracture of the right ramus of the lower jaw between the first bicuspid and the cuspidatus, apparently about as perfect and complete as though it had been cut through with a saw, although the result of a blow from the fist at an Irish knock-down frolic.

After considering the ill adaptation and awkwardness of the appliances recommended as splints in such cases, I conceived the idea of taking an impression in wax of the teeth in the lower jaw on the injured side while the fractured parts were held together in position; and also an impression of the upper teeth on the corresponding side; then making two silver plates of the exact form of the teeth, fitting over the crowns—thus making, by fastening them together, a perfectly fitting splint. I did so; after getting the impressions I filled them with a mixture of calcined plaster and water, mixed to the consistency of cream. Thus I obtained models of both jaws with their teeth; from them I made metallic dies; and upon them I had the silver plates molded to the exact forms required.

These plates, well trimmed and fettered, I placed in the patient's mouth, with some wax intervening. Then I brought up the under jaw into its normal position, and approximated it to the upper to within about half an inch. Then opening the mouth, I took out the plates adhering to each other by the wax in just the relative position desired. Investing them in a mixture of plaster and water, the wax was removed, and the plates were fastened together by soldering the posterior extremities; and near the anterior ends a bar was soldered across between them, thus fastening both plates together firmly.

With this splint placed in the mouth, covering the crowns of the teeth, the fractured parts of lower jaw were adjusted by a bandage over the head, and so readily were they retained in position that the bandage was easily renewed, without danger of displacement. The jaw was kept in situ until it was fully united.

The union was complete without the slightest deformity. There was an incomplete anchylosis on removing the splint, which alarmed the patient, until I assured him that he would soon acquire again full as free use of his jaw as he would wish to have. He is now in the army of the Union, perhaps to have it broken again. This arrangement answered perfectly all the other indications required. Nourishment can be readily administered—there is little hindrance to the freedom of motion of the tongue, and it affords (which is very important for the comfort of the patient), good facilities for cleansing the mouth by a free use of the syringe and the tooth-brush.

Such a splint would be equally as useful in some cases of fracture of the upper jaw.

These splints, to fit accurately, must be made for each case. They can be made by any surgeon, if he has much ingenuity (and he must have, to be a good surgeon), in almost every camp hospital. In place of silver or gold plate, which he *should* have in his medical stores, he can use the block tin of a tea-pot or a tumbler. Or for metal to make his metallic dies, he can melt down some of the same, or some musket-balls.—*Med. and Surg. Reporter.*

